# Network analysis of comorbid depression, suicidality and biomarkers on HPA axis among mood disorder patients to psychiatric emergency services

**DOI:** 10.1038/s41398-023-02503-5

**Published:** 2023-06-14

**Authors:** Yi-Fan Wang, Guang-yun You, Tian Han, Yi Liu, Juan Li, Xiao Ji, Xiao-meng Xie

**Affiliations:** 1 Beijing Key Laboratory of Mental Disorders, National Clinical Research Center for Mental Disorders & National Center for Mental Disorders, Beijing Anding Hospital, & The Advanced Innovation Center for Human Brain Protection, Capital Medical University, Beijing, China; 2 Department of Psychiatry, The People’s Hospital of Juxian County, Juxian, 276500 China

**Keywords:** Predictive markers, Human behaviour

## Abstract

Rapid assessment and intervention of suicide risk are common and challenging in psychiatric emergency departments (PED). It is unclear whether distinct pathophysiological processes exist among depressive patients with suicidality. This study examined the network structures of biomarkers on Hypothalamic-Pituitary-Adrenal (HPA) axis, such as Adrenocorticotropic hormone (ACTH) and Corticosterone (Cort), as well as suicidality and depressive symptoms in mood disorder patients in PED. Mood disorder patients in PED were assessed with the measurements of suicidality and depressive symptoms, respectively. A network analysis was performed to identify central symptoms and bridge symptoms of this network and their links to ACTH and Cort. Network stability was examined using the case-dropping procedure. The Network Comparison Test (NCT) was conducted to evaluate whether network characteristics differed by gender. A total of 1815 mood disorder patients were recruited. The prevalence of SI was 31.2% (95% CI: 28.15–34.21%), SP was 30.4% (95% CI: 27.39–33.41%), SA was 30.62% (95% CI: 27.61–33.64%) among psychiatric outpatients. The mean score of HAMD-24 was 13.87 ± 8.02. Network analysis revealed that ‘Somatic anxiety’ had the highest expected centrality, followed by ‘Hopelessness’ and ‘Suicide attempt’. ‘Corticosterone’ and ‘Retardation’ may be the main bridge symptoms between depressive symptoms and the suicidality community. The network model showed a high degree of stability. Gender did not significantly influence the network structure. The central symptoms and key bridge symptoms identified could be potential targets for interventions of the HPA axis, which is designed for regular screening of a range of suicidal activity. In the light of this, timely treatment should be provided for psychiatric emergency care.

## Introduction

Suicide is an important cause of death worldwide. Approximately 800,000 people per year die by suicide, accounting for about 1.5% of all deaths [1]. It is estimated that one in ten of them had been admitted to an emergency department within two months of their death [2]. Since the emergence of ideation, attempts and completed suicide are associated with many factors, such as the demographic, psychosocial, clinical and biological factors [3, 4]. In addition, as a promising setting for suicide prevention, the psychiatric emergency department (PED) needs to establish some appropriate biological markers for universal screening, risk assessment and follow-up care standards [5].

Hyperactivity of the Hypothalamic–Pituitary–Adrenal (HPA) axis is well documented among patients suffering from psychiatric disorders [6]. Previous studies provide evidence that the Hypothalamic–Pituitary–Adrenal (HPA) axis is hyperactivity in young adult mood disorder inpatients who manifesting suicidal behavior and ideation [7, 8]. Furthermore, the lowest plasma cortisol and corticotrophin responses were found in the patients who recently attempted suicide. Also, a previous study suggests that the usage of neuroendocrine testing of HPA axis functioning as a complementary tool in suicide prevention [9].

Depression is the most common psychiatric disorder in people who die by suicide [10] and, therefore, detection of individuals at risk of suicide, while clearly extremely important, can be difficult. Many previous studies indicated that there is a positive association between severity of depression and suicidality [11, 12] but the specific item association is poorly understood. A two-year prospective cohort study following 91 individuals with mood disorders has found higher suicidality to be more strongly correlated with depression and hopelessness with the scale of HAMD (Hamilton Rating Scale for Depression) [13]. Furthermore, a flow network analysis result shows that suicide ideation was seems to be related directly to loneliness, sadness, pessimism, feeling important to their family, self-hatred and self-blame [14].

Traditionally, previous studies found that the total score of the depression positively correlated with the HPA axis, but the item level of the association is not clear. In addition, comorbidity depression and suicidality rely on total scale scores to describe symptom severity in the present researches. Unfortunately, such approach may obscure meaningful connections between individual symptoms. Network analysis has been more and more widely applied in the field of psychology and psychiatry in recent years. Network analysis is a novel analytical technique used to gain insights into a biological system by predicting how multiple syndromes and biological indicators [15, 16]. Network analysis has been used to elucidate structural relationships in symptoms in dimensions such as emotional, behavioral, and cognitive symptoms to screen for the most influential symptoms in the disease [17–19]. The clinical features of depression and suicidality are closely related to neuroendocrine factors. Therefore, findings based on inpatients and community samples do not necessarily as applicable within stressful contexts of rapidly changing psychiatric emergency departments [20, 21]. To the best of our knowledge, no study has examined comorbid ACTH and Cort, suicidality, and depressive symptoms from the perspective of network analysis.

Using a network approach, this study investigated the inter-relationships between ACTH and Cort, suicidality, and depressive symptoms in a large representative sample of mood disorder patients. The main focus was upon identifying central symptoms and bridge symptoms within this network model. The biomarkers were evaluated for association with symptoms simultaneously, leading to greater insights into the pathophysiology of disease states.

We hypothesize that high levels of Cort may represent an increase in neurobiological activity which might contribute to suicidality by exacerbating the level of hopelessness in depression that might hinder the foresight and consideration of consequences. If this hypothesis is correct and clinicians have sufficient evidence to identify the key risk factors for suicide in individuals with depression is therefore essential if clinicians are to identify those most at risk and intervene appropriately, as there is good evidence that monitoring and active treatment in high-risk patients may result in reduced suicide rates [22].

## Methods

### Patients and study sites

There was a cross-sectional survey carried out between January 2021 and March 2022, in Department of Emergency in Beijing Anding Hospital, which is the only 24-hour emergency service for psychiatric hospitals in Beijing municipality and neighboring provinces in North China. To be eligible, all participants were: (1) outpatients receiving emergency maintenance treatment for a major psychiatric disorder; (2) provided written and informed consent; (3) patients diagnosed with mood disorders (F30–F39), according to the International Statistical Classification of Diseases and Related Health Problems-10th revision (ICD-10) diagnostic criteria established by two consultant psychiatrists with over 15 years clinical experience; (4) and presenting with depression symptoms (i.e., 24-items Hamilton Depression Rating Scale (HAMD-24) total score of ≥8 [23], understanding of the aims of the study and the contents of the clinical interview and willingness to provide informed consent. This study was approved by the ethical committees of the Beijing Anding Hospital.

### Blood collection and assays of ACTH, Cort

Serum samples were collected from all patients between 7:30 AM and 8:30 AM. Controlling for age and gender, the levels of 8 (7.30–8.30) AM serum were assayed by ACTH and Cort (μg/dl) using chemiluminescence. The laboratory personnel were blind to all clinical information. Samples and data were processed following standard operating procedures with the appropriate approval of the Ethics and Scientific Committee of Capital Medical University.

### Measurement

In this study, the current suicidal ideation, suicide plan and suicide attempts were assessed through a face-to-face interview, which was conducted by a trained attending psychiatrist within 12 hours after admission. Following previous studies (Li et al., 2017), SI was assessed with a standard question (“Have you thought that you would be better off dead currently?”) that included a binary response option (yes/no). SP were assessed with a standard question (“Have you made a plan for suicide currently?”) featuring a binary response option (yes/no). SA were evaluated with a standard question (“Have you attempted suicide currently?”), including a binary response option (yes/no). Depressive symptoms was assessed using the validated Chinese version of the 24-items Hamilton Depression Rating Scale (HAMD-24) [24]. The Scale had been validated in the Chinese population with a sensitivity of 0.87 and specificity of 0.92 [25]. Each item with a score between 0 and 4 of the HAMD-24. A total score of 8 or above was diagnosed as depression, with a higher score indicating more severe depressive symptoms [23]. Following previous studies [26], item HAMD3 (“Suicide ideation”) is redundant with the SI component of suicidality; therefore, it was not included in network analysis.

### Statistical analyses

The network model of depression, suicidality and ACTH was computed using the R software [27]. We computed the polychoric correlations between all the items to investigate the edges of the network, and also estimated the Graphical Gaussian Model (GGM), with the graphic least absolute shrinkage and selection operator (LASSO) and Extended Bayesian Information Criterion (EBIC) model using the R package ‘*graph*’ [28].

The importance of each node in the network was examined by estimating centrality indices of the network structure with the R package *‘graph*’ [29]. Specifically, the centrality index of expected influence (EI) was computed for each node in the network (i.e., the sum of the weights of the connections, in absolute value), because EI is the most stable and interpretable centrality index [28]. Additionally, previous studies [30, 31] on comorbid psychiatric syndromes found that ‘ACTH’ was commonly reported to link between different symptom communities as a key node. Therefore, the node-specific predictive betweenness of ‘ACTH’ (i.e., how often a node lies on the pathways between two other nodes, always with the ‘ACTH’ node as either of them across 1000 nonparametric bootstrap iterations) was estimated [32–34]. To identify particular symptoms that were directly associated with ACTH, the ‘flow’ function in the R package ‘*qgraph’* was used [28].

In particular for the stress axis, factors such as gender and sex steroids regulate ACTH secretion strongly in animals, investigations in human subjects are both limited and discrepant [35–37]. Considering the moderating effects of gender, network models were compared between genders. Following previous studies [38, 39], the differences in network characteristics between male and female participants were compared using the R ‘*NetworkComparisonTest’* package (Version 2.2.1) [40] with 1000 permutations. The difference in network structure (e.g., distributions of edge weights), global strength (e.g., total absolute connectivity among the symptoms), and each specific edge between subsamples (i.e., females vs. males) were also examined.

### Missing data

To minimize the likelihood of any missing values, researchers in each participating hospital checked the completion of the relevant questionnaires prior to data submission, and reminded participants to complete the missing items at a voluntary basis. As network methodologies are currently incompatible with multiple imputed data sets, network analysis in this study was performed based on the dataset comprising participants who completed the HAMD-24.

## Results

A total of 1815 patients were invited to participate in this study, of whom 898 met the study criteria and were included in analyses. The prevalence of SI was 31.2% (95% CI: 28.15–34.21%), SP was 30.4% (95% CI: 27.39–33.41%), SA was 30.62% (95% CI: 27.61–33.64%) among psychiatric outpatients. The mean score of HAMD-24 was 13.87 (Standard Deviation (SD):8.02). Data reating to demographics and the clinical characteristics of the study population are summarized in Table 1.

**Table 1:** The social-demographic and clinical characteristics.

Variables	Total	Male	Female
	N (%)	N (%)	N (%)
Occupation	782 (87.1)	320 (87.4)	462 (86.8)
Urban	208 (23.2)	90 (24.6)	118 (22.2)
Medical insurance	647 (72.0)	263 (71.9)	384 (72.2)
Unmarried	398 (44.3)	154 (42.1)	244 (45.9)
Family history	238 (26.5)	94(25.7)	144 (27.1)
Good health status	699 (77.8)	281 (76.8)	418 (78.6)
Poor relationship with friends/family members	376 (41.9)	142 (38.8)	234 (44.0)
Stress event	143 (15.9)	54 (14.8)	89 (16.7)
	**Mean (SD)**	**Mean (SD)**	**Mean (SD)**
Age (years)	37.22 (16.99)	39.51(17.57)	35.64 (16.42)
BMI	24.33(14.74)	25.91(18.95)	23.25 (10.82)
Education background (years)	13.08 (3.66)	13.17 (3.47)	13.02 (3.80)
Illness duration (years)	7.55 (8.54)	7.75 (9.13)	7.41 (8.11)
First episode age (years)	29.88 (15.05)	31.97 (15.98)	28.44 (14.22)
Cortisone (nmol/L)	16.68 (11.41)	17.10 (13.67)	16.39 (9.55)
ACTH (pg/ml)	31.84 (28.77)	37.31 (30.72)	28.07 (26.74)
HAMD-24 total	13.87 (8.02)	13.32 (8.18)	14.11 (7.91)

### Network structure

**Fig. 1:**
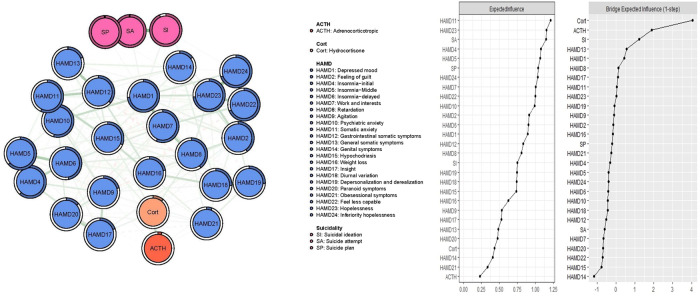
Network analysis of comorbid depression, suicidality and ACTH among mood disorder patients.

Figure 1 presents the network structure of comorbid suicidality, depression symptoms, ACTH and Cort in participants with mood disorders. The network model showed that the connection HAMD4 (‘Insomnia-initial’) – HAMD5 (‘Insomnia-middle’) was the strongest positive edge in the depressive symptom, followed by HAMD10 (‘Psychiatric anxiety’) – HAMD11 (‘Somatic anxiety’), and HAMD18 (‘Diurnal variation’) – HAMD19 (‘Depersonalization and derealization’). In the suicidality community, SA (‘Suicide attempt’) - SP (‘Suicide plan’) were the strongest edge, followed by SA (‘Suicide attempt’) - SI (‘Suicidal ideation’).

**Fig. 2:**
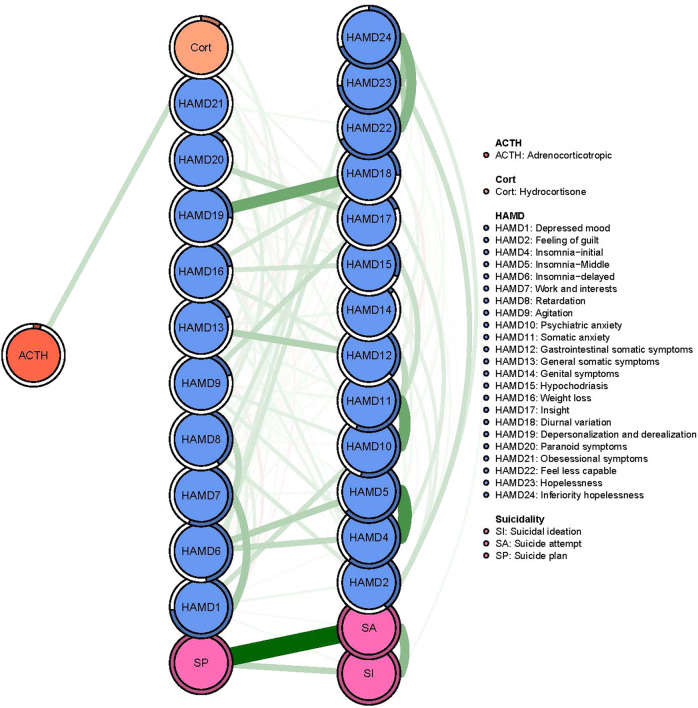
Flow network of ACTH.

In terms of EI, the node HAMD11 (‘Somatic anxiety’) had the highest EI centrality, followed by HAMD23 (‘Hopelessness’) and SA (‘Suicide attempt’) in the network (Fig. 1). In terms of bridge EI, Cort (‘Corticosterone’) was the most key bridge symptom linking the suicidality and depressive symptom, followed by ACTH (‘Adrenocorticotropic’) and SI (‘Suicidal ideation’) (Fig. 1). In addition, we found that Cort (‘Corticosterone’) had the strongest positive association with ACTH in the flow network model, followed by the HAMD13 (‘General somatic symptoms’) and HAMD9 (‘Agitation’) (Fig. 2).

**Fig. 3:**
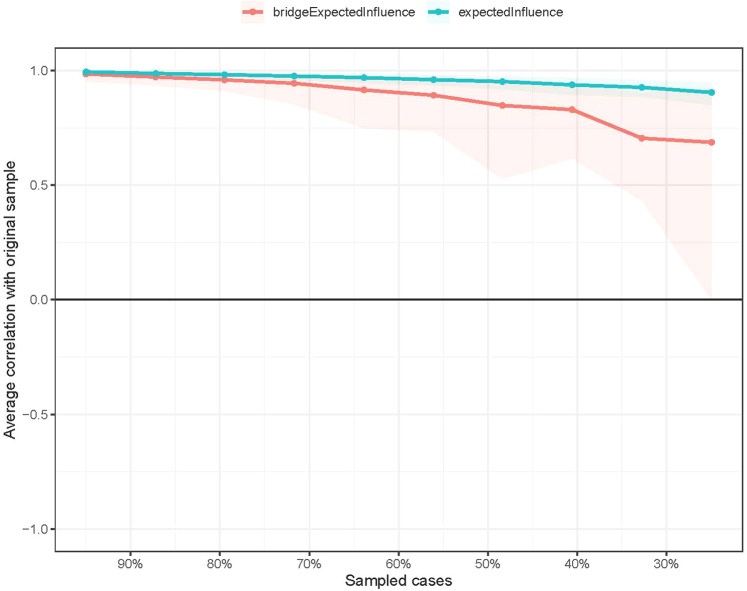
The stability of centrality and bridge centrality indices using case-dropping bootstrap.

For the network stability, the centrality of EI had an excellent level of stability (i.e., CS-coefficient = 0.75 (95% CI: 0.675-1)), which indicates that 75% of the sample could be dropped, and the structure of the network did not significantly change (Fig. 3). The bootstrap difference test shows that most comparisons between edge weights are statistically significant.

### Node-specific predictive betweenness measure

**Fig. 4:**
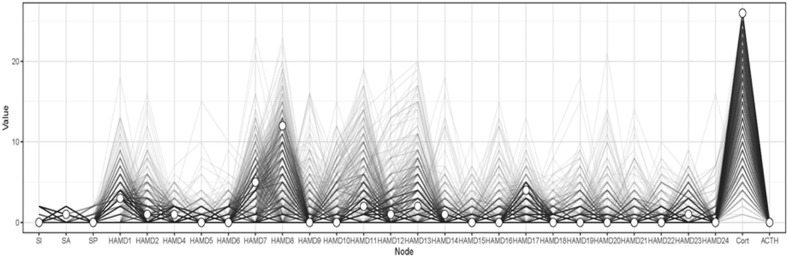
Node-specific predictive betweenness.

Previous studies found that ‘ACTH’ was strongly associated with the development of depression symptoms and suicidality in both patients with mood disorders [31]. The bootstrapping procedure showed that the estimation of node-specific predictive betweenness (i.e., items that more often lie on the shortest pathways from ‘ACTH’ to other nodes) was considerably less precise than that of other features of the network. Figure 3 shows the node-specific predictive betweenness values for each node in the network. The white dots represent the node-specific predictive betweenness in the study sample, while the black lines represent the variability of the measure across 1000 nonparametric bootstrap iterations. Cort (‘Corticosterone’) had the highest node-specific predictive betweenness score, followed by HAMD8 (‘Retardation’). This finding suggests that ‘Corticosterone’ and ‘Retardation’ may be the main bridge symptoms between depressive symptom and the suicidality community (Fig. 4).

### Network comparison tests by gender

The comparison of network models by gender did not find significant differences in network global strength (network strength: 11.37 in female participants; 11.40 male participants; M = 0.214, *P* = 0.571) and edge weights (S = 0.026, *P* = 0.969, Supplementary Fig. S1–S4).

## Discussion

This study is the first study conducted in a PED in China to examine comorbidity between levels of ACTH, Cort and depression and suicidality symptoms using network analysis. In the observed network model, depressive symptoms (e.g., ‘somatic anxiety’ and ‘hopelessness’) and suicide attempt were more influential in the sample and Corticosterone acted as the most key bridge symptom linking the suicidality and depressive symptom, followed by ACTH and SI. Gender was not a significant influence for the network structure, and the network had a high degree of stability.

In this network, we found that the strongest edge in the whole network was between depressive symptoms HAMD4 ‘Insomnia-initial’ and HAMD5 ‘Insomnia-middle’. This diverges from results of a major depression population study exploring relations between depressive symptoms and quality of life wherein the edge HAMD1- HAMD2 (Depressive mood - Feeling of guilt) was strongest [41]. Specifically, our study was conducted in an emergency room setting, leading to more extensive difficulty falling asleep and staying asleep due to acute stress states [42]. Our findings are consistent with previous findings, indicating hopelessness was identified as one of the central depressive symptoms in patients brought to the emergency room due to suicidality attempt [43]. The pessimism that grows out of hopelessness may result from adverse life events, such as bereavement [44, 45], economic and social problems [46], and physical illnesses [47, 48]. Stressors, medical and psychiatric comorbidities may trigger the progression of hopelessness to SI and suicidal behavior. It’s a continuous process, that is, from active suicidal intent, to planning, and finally to suicidal behavior [49]. The severity and urgency of suicidality must be dealt with by an emergency psychiatric department rather than a general psychiatric clinic [50]. The network identified in this study also showed that the node HAMD11 ‘Somatic anxiety’ was another central symptom of depression and suicidality symptoms network model. Our findings also confirmed the previous findings that somatic anxiety of anxiety cluster was significantly associated with youth-reported SI [51–53]. It has been hypothesized that chronic exposure to somatic anxiety may lead to the consideration of suicide as a means of relieving symptoms [54] based on escape-based mentality theory. Consistent with this theory, individuals suffering symptoms of somatic anxiety are more likely to have suicidal thoughts and behaviors due to an escape-based mentality that give an idea of escape from the situation and reduce mental pain (Williams & Pollock, 2000).

The strong association between hopelessness and suicidality suggests an underlying neurobiology, manifested by an increased stress hormone response, which was in line with extant literature [55]. Furthermore, the epigenetic mechanisms had indicated that glucocorticoid receptor (GR) downregulation and increased sensitivity to GR are present in suicide attempters, resulting in decreased HPA axis activity during acute depressive states [31, 56]. The blunted stress hormone response was a trait of suicide risk. The patients with a history of suicide attempts belonged to a subgroup of individuals that exhibit a more attenuated HPA response [57]. As previously reported, low baseline cortisol levels in suicide attempters had been interpreted to be associated with prolonged chronic stress leading to the “ burnt-out “ of the most important HPA axis, resulting in a suicide attempt [58, 59]. The trophic effects of ACTH on the adrenal gland induced by emotional symptoms, and elevated cortisol in SI, were associated with chronic increases in ACTH and decreased negative feedback compared with SA.

In particular, ‘Cort’ acted as both an important central symptom and bridge symptom in the current network model. ‘Cort’ and ‘Retardation’ also had high node-specific predictive betweenness (i.e., more often lies on the shortest pathway from ACTH to other nodes). These results are consistent with previous research identifying that elevated corticotropin-releasing hormone (CRH) peptides in the cerebrospinal fluid were detected in suicidal patients [60], as well as increased CRH mRNA expression in the prefrontal cortex [61] which are associated with mental stress. Dysfunctions of the prefrontal cortex leads to dysregulation of emotional and cognitive responses, leading to strong and long-term negative emotional states and SI [62]. The activity of HPA axis can be signficantly affected by the diurnal variation. Salivary Crot levels over the day (between 8 AM and 10 PM) have been shown to correlate negatively with performance in hippocampus-related neuropsychological memory tasks and executive function [63]. It was reported that the different threshold for the Crot levels may offer a better identification of suicide in the dexamethasone suppression test (DST) in patients with mood disorders [64]. Other HPA axis measures, such as the cortisol awakening response (CAR) and diurnal cortisol slope, have also been associated with impaired cognition in depressive patients [65]. Cognitive responses change in mental stress environment often manifests as retardation, triggering suicidal behavior [66–68].

Identifying central symptoms and bridge symptoms within the suicidality and depressive symptom network model has possible clinical significance; targeting these symptoms may contribute to prevention among at-risk suicidality patients [69]. This network model highlighted the important role of control processes for possible targeted treatment for mood disorders patients in PED at high risk of suicide. The Safety Planning Intervention (SPI) is a promising intervention to target central symptoms and bridge symptoms including “somatic anxiety”, “hopelessness”, and “suicide attempt” via a prioritized list of coping skills and strategies and mitigate risk of suicide when evaluating and treating PED patients who are at increased risk for suicide [70, 71]. 6 months following discharge is a defined high-risk period for suicide, and follow-up SPI are recommended for patients with a history of suicide in the PED [72].

Strengths of this study included the relatively large sample size, use of standardized assessment tools, and application of advanced network analysis statistics designed to explore the structure of ACTH and suicide model in mood disorder patients which could identify the potential targets in treatments and preventions for mood disorder patients in psychiatric emergency with these problems. However, our study has several methodological limitations to consider: Firstly, causal relations between variables could not be determined due to the cross-sectional nature of the study. Future, longitudinal studies are warranted to explain the temporal causal relationships between ACTH and suicide in mood disorders patients. Secondly, suicide community in our study were assessed by self-report measures. Therefore, the possibility of recall bias could not be excluded. Thirdly, patients with clinically diagnosed mood disorders including bipolar and unipolar depression were included, thus the heterogeneity of study participants may contribute to estimation of network structure for the variables. Fourthly, other moderators such as age, education level, and other clinical information should be included in the network comparison analyses. In addition, in order to ensure the rhythm changes of HPA, blood samples have been collected in the morning. Lastly, a consecutive sample of patients from only one major PED in China was recruited, which limits the generalization of the findings to other psychiatric settings.

In conclusion, symptoms related to somatic anxiety and hopelessness were identified as those most critical within the depression-suicidality network model in terms of node and bridge centrality as well as their associations with ACTH and Cort among mood disorders patients in emergency department. As such, specific depressive symptoms as potential targets for intervention can warrant the physicians’ attention in the evaluation protocol. In light of this, timely treatment should be provided for those seeking emergency psychiatric help.

## Supplementary information

Supplementary materials

## Data Availability

The data of the investigation will be made publicly available if necessary.
