# Supplementary material for: Network analysis of comorbid depression, suicidality and biomarkers on HPA axis among mood disorder patients to psychiatric emergency services
Source: Transl Psychiatry. 2023 Jun 14;13:203. doi: 10.1038/s41398-023-02503-5 (PMC10267111; doi:10.1038/s41398-023-02503-5)

**Supplementary materials**

Figure S1. Estimated network model for comorbid depression, suicidality and ACTH among in females and males

Figure S2. Comparison of network flow of ACTH between females and males

Figure S3. Comparison of Node-specific predictive betweenness in females and males

Figure S4. Comparison of network properties between females and males

Table S1. Means, standard deviations, skewness, and kurtosis

| Item | M | SD | Skewness | kurtosis |
| --- | --- | --- | --- | --- |
| SI | 0.31 | 0.46 | 0.82 | -1.34 |
| SA | 0.31 | 0.46 | 0.84 | -1.29 |
| SP | 0.30 | 0.46 | 0.85 | -1.27 |
| HAMD1 | 1.10 | 1.32 | 0.56 | -1.44 |
| HAMD2 | 0.25 | 0.65 | 2.83 | 7.91 |
| HAMD4 | 1.17 | 1.04 | 0.26 | -1.19 |
| HAMD5 | 1.13 | 1.01 | 0.24 | -1.22 |
| HAMD6 | 0.80 | 0.94 | 0.72 | -0.80 |
| HAMD7 | 0.87 | 1.11 | 0.79 | -0.92 |
| HAMD8 | 0.39 | 0.76 | 1.84 | 2.33 |
| HAMD9 | 0.93 | 1.11 | 0.73 | -0.89 |
| HAMD10 | 0.88 | 1.00 | 0.68 | -0.85 |
| HAMD11 | 0.56 | 0.87 | 1.38 | 0.73 |
| HAMD12 | 0.24 | 0.60 | 2.51 | 5.47 |
| HAMD13 | 0.09 | 0.42 | 5.16 | 27.13 |
| HAMD14 | 0.06 | 0.35 | 6.60 | 46.14 |
| HAMD15 | 0.13 | 0.49 | 4.25 | 18.35 |
| HAMD16 | 0.12 | 0.46 | 4.13 | 17.65 |
| HAMD17 | 1.88 | 1.17 | -0.54 | -1.07 |
| HAMD18 | 0.06 | 0.33 | 6.04 | 40.16 |
| HAMD19 | 0.10 | 0.42 | 5.03 | 28.20 |
| HAMD20 | 0.53 | 0.93 | 1.58 | 1.29 |
| HAMD21 | 1.07 | 0.35 | 6.36 | 46.56 |
| HAMD22 | 0.60 | 0.96 | 1.25 | 0.11 |
| HAMD23 | 0.59 | 1.00 | 1.41 | 0.51 |
| HAMD24 | 0.50 | 0.90 | 1.58 | 1.16 |
| Cort | 1.13 | 0.56 | 0.03 | 0.00 |
| ACTH | 1.22 | 0.42 | 1.34 | -0.21 |

Figure S1. Estimated network model for comorbid depression, suicidality and ACTH among in females and males
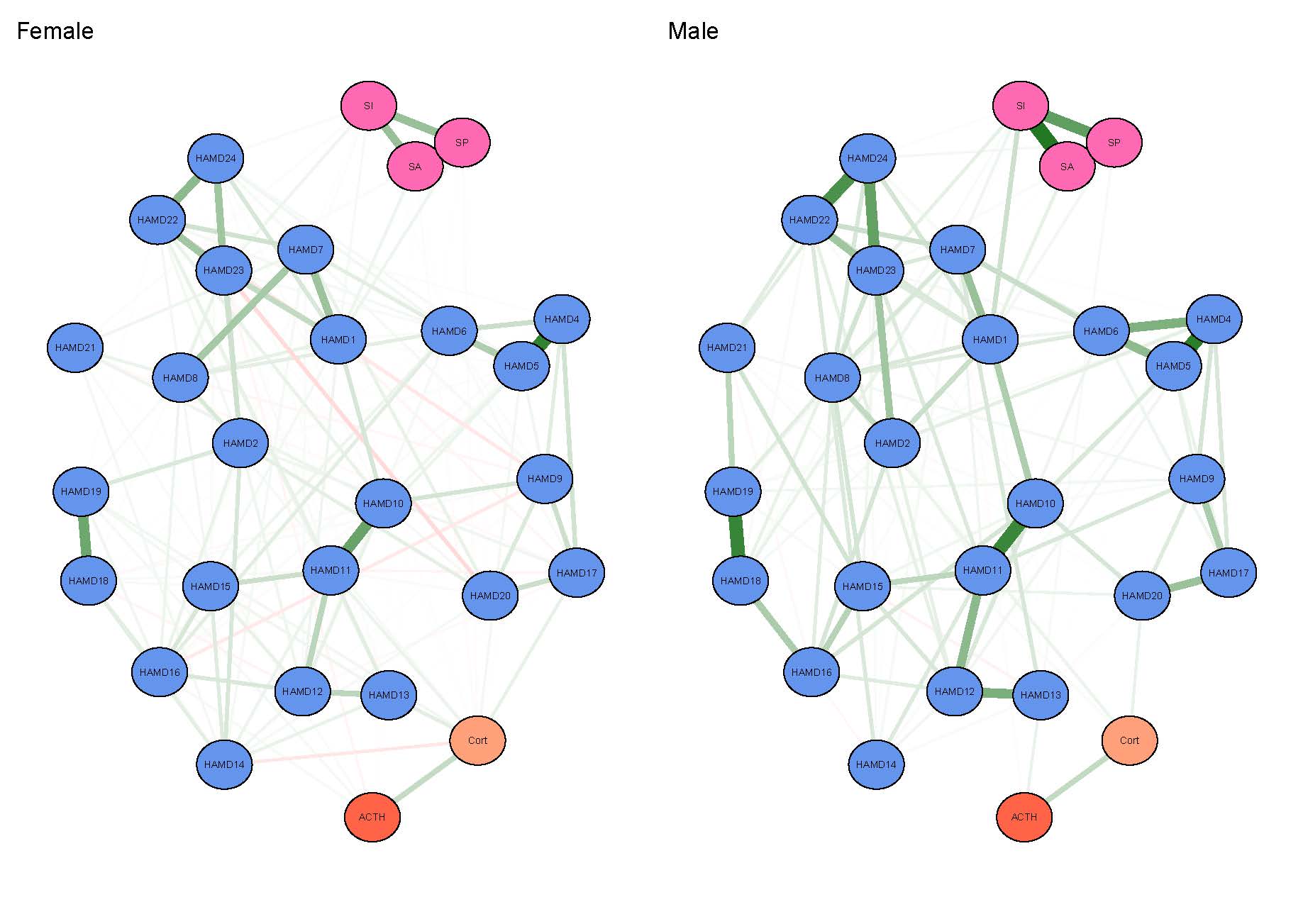


Figure S2. Comparison of network flow of ACTH between females and males


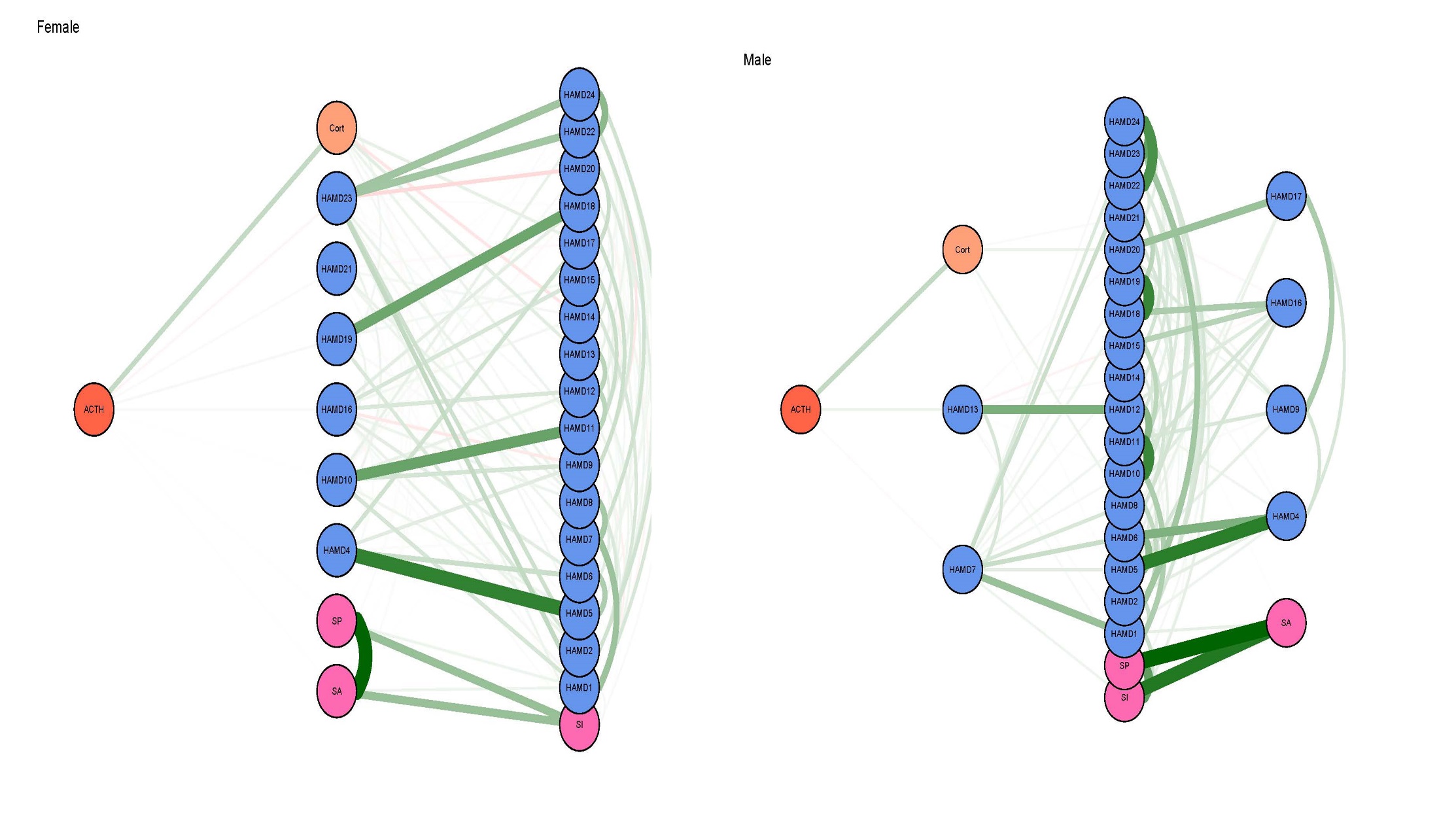


Figure S3. Comparison of Node-specific predictive betweenness in females and males


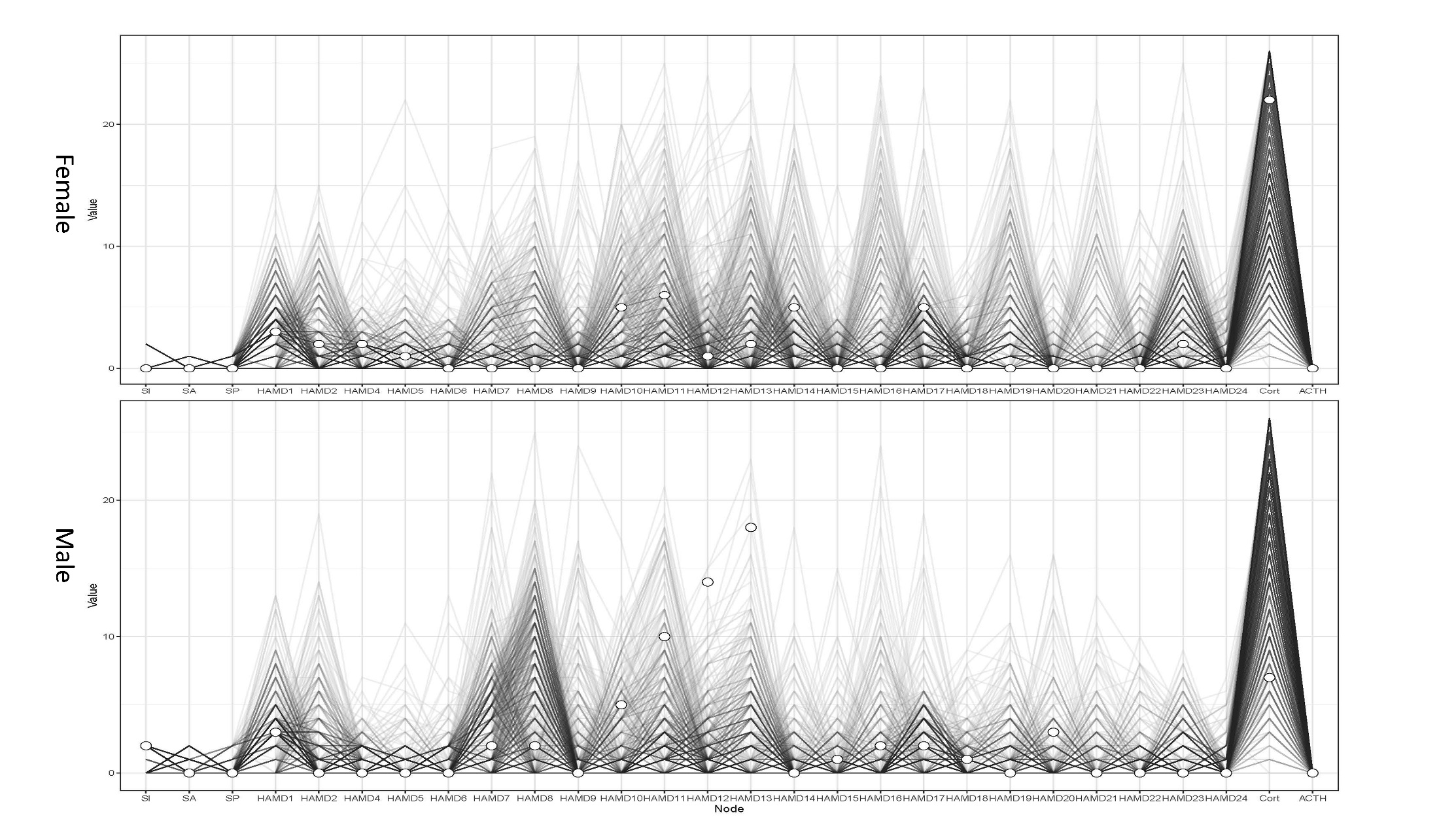


Figure S4. Comparison of network properties between females and males


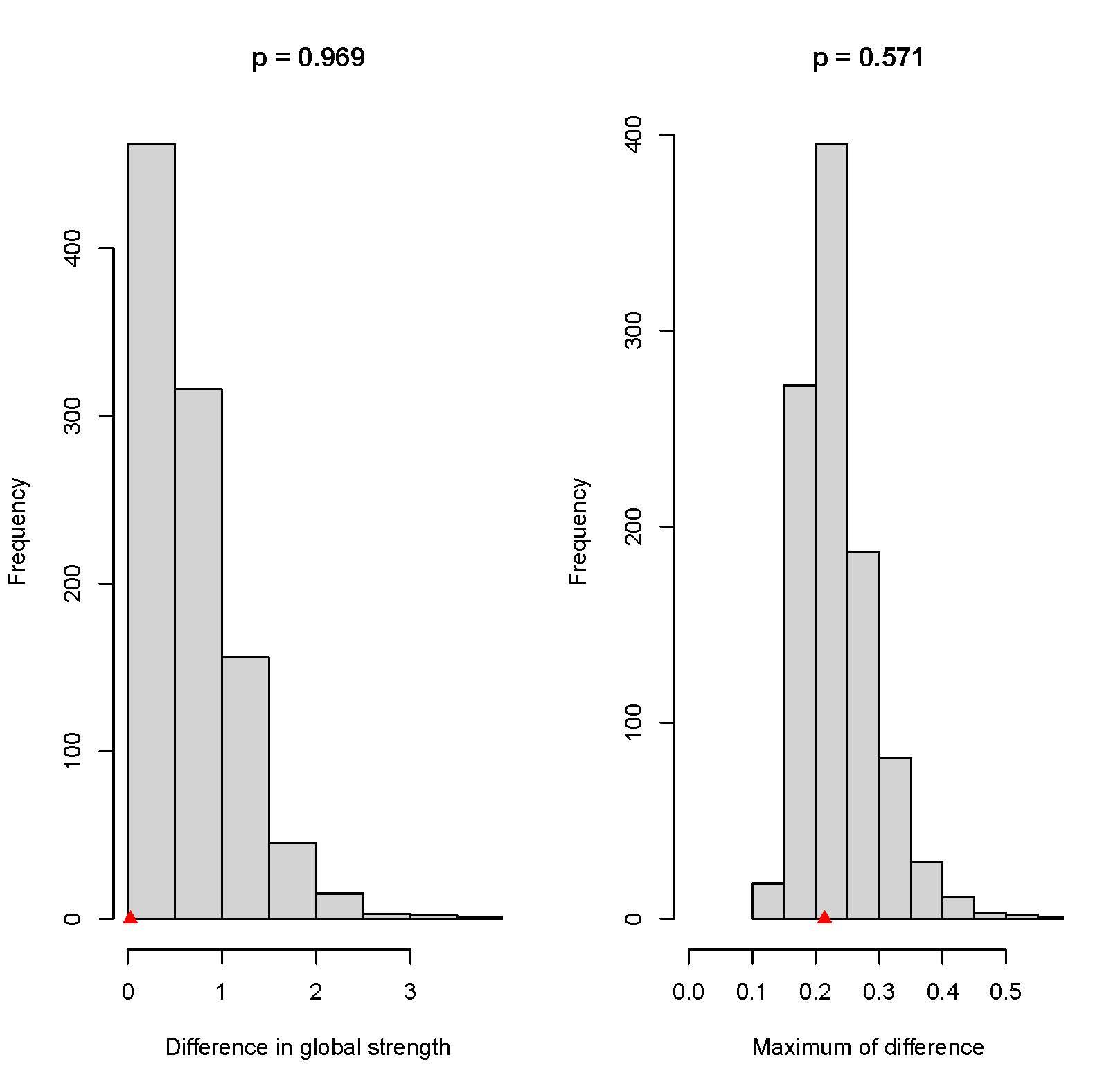

Supplement: Supplementary file 1 — Supplementary materials [file 41398_2023_2503_MOESM1_ESM.docx]
